# Nuclear DNA segments homologous to mitochondrial DNA are obstacles for detecting heteroplasmy in sugar beet (*Beta vulgaris* L.)

**DOI:** 10.1371/journal.pone.0285430

**Published:** 2023-08-08

**Authors:** Eigo Taniguchi, Kosuke Satoh, Megumi Ohkubo, Sachiyo Ue, Hiroaki Matsuhira, Yosuke Kuroda, Tomohiko Kubo, Kazuyoshi Kitazaki

**Affiliations:** 1 Research Faculty of Agriculture, Hokkaido University, Sapporo, Hokkaido, Japan; 2 Hokkaido Agricultural Research Center, National Agriculture and Food Research Organization, Memuro, Hokkaido, Japan; ICAR Research Complex for North Eastern Hill Region Manipur Centre, INDIA

## Abstract

Heteroplasmy, the coexistence of multiple mitochondrial DNA (mtDNA) sequences in a cell, is well documented in plants. Next-generation sequencing technology (NGS) has made it feasible to sequence entire genomes. Thus, NGS has the potential to detect heteroplasmy; however, the methods and pitfalls in heteroplasmy detection have not been fully investigated and identified. One obstacle for heteroplasmy detection is the sequence homology between mitochondrial-, plastid-, and nuclear DNA, of which the influence of nuclear DNA segments homologous to mtDNA (numt) need to be minimized. To detect heteroplasmy, we first excluded nuclear DNA sequences of sugar beet (*Beta vulgaris*) line EL10 from the sugar beet mtDNA sequence. NGS reads were obtained from single plants of sugar beet lines NK-195BRmm-O and NK-291BRmm-O and mapped to the unexcluded mtDNA regions. More than 1000 sites exhibited intra-individual polymorphism as detected by genome browsing analysis. We focused on a 309-bp region where 12 intra-individual polymorphic sites were closely linked to each other. Although the existence of DNA molecules having variant alleles at the 12 sites was confirmed by PCR amplification from NK-195BRmm-O and NK-291BRmm-O, these variants were not always called by six variant-calling programs, suggesting that these programs are inappropriate for intra-individual polymorphism detection. When we changed the nuclear DNA reference, a numt absent from EL10 was found to include the 309-bp region. Genetic segregation of an F_2_ population from NK-195BRmm-O x NK-291BRmm-O supported the numt origin of the variant alleles. Using four references, we found that numt detection exhibited reference dependency, and extreme polymorphism of numts exists among sugar beet lines. One of the identified numts absent from EL10 is also associated with another intra-individual polymorphic site in NK-195mm-O. Our data suggest that polymorphism among numts is unexpectedly high within sugar beets, leading to confusion about the true degree of heteroplasmy.

## Introduction

Heteroplasmy refers to the coexistence of multiple mitochondrial DNA sequences in a cell [1]. The causes of heteroplasmy may include mutation, paternal leakage of mitochondrial DNA (mtDNA) [2], or possibly cell-to-cell movement of mitochondria through grafting [3].

Heteroplasmy seems an unlikely event because mitochondrial transmission includes several anti-heteroplasmic processes, such as active degradation of paternal mitochondria or paternal mtDNA, somatic sorting out (vegetative segregation), and germline bottlenecks [4]. Moreover, heteroplasmy *per se* may be deleterious and counter-selected because heteroplasmic individuals can suffer a fitness penalty even though the coexisting mtDNAs have no effect when they are homoplasmic [5]. Nevertheless, it is surprising that heteroplasmy is reported in many eukaryotes [6–8]. Heteroplasmy has been proposed to play an evolutionary role by maintaining mtDNA polymorphism within a population [1, 2].

In plants, some mitochondrial mutations have been associated with heteroplasmy; examples have been reported in maize (*Zea mays*) [9], cucumber (*Cucumis sativus*) [10], Arabidopsis (*Arabidopsis thaliana*) [11] and woodland tobacco (*Nicotiana sylvestris*) [12]. A common bean (*Phaseolus vulgaris*) male-sterile mutant caused by specific mitochondria is heteroplasmic with male sterility-inducing and noninducing mitochondria; the plant restores male fertility when normal mitochondria predominate [13]. A similar phenomenon was reported in pearl millet (*Pennisetum glaucum*) [14]. Heteroplasmy has also been observed in plants with no apparent phenotype. For example, the stoichiometric ratio of different mtDNA molecules changes significantly when tobacco (*N*. *tabacum*) plants regenerate from callus [15].

With the remarkable advances in nucleotide sequencing technology (next-generation sequencing (NGS)), it is possible to obtain a comprehensive view of cellular DNA sequences [16]. Thus, such a high-throughput technique could be useful in detecting heteroplasmy [2] in combination with variant caller software initially developed for identifying nuclear DNA (nuDNA) polymorphisms. However, it is questionable whether variant callers recognize potential heteroplasmic sites as variants or dismiss them as sequencing errors because the amount of heteroplasmic variant DNA molecules is usually low.

Recent reports increasingly claim that heteroplasmy is a common state in plants; plant mitochondria contain DNA molecules that differ from their siblings with single nucleotide polymorphic sites (SNP), small deletions or insertions (indels), or DNA recombination [6, 17, 18]. Heteroplasmic variant mtDNA has also been reported to be inherited paternally [17, 18]. Some of these reports were based solely on detecting small amounts of variant DNA molecules resembling mtDNA. Interpreting such data as a manifestation of heteroplasmy assumes that all mtDNA-like sequences only exist in mitochondria. This assumption should be examined in detail.

The size of plant mtDNA varies but typically ranges from 200 to 500 kbp, a much larger size than found in vertebrates (~16 kbp) [19]. The large size can be explained by the expansion of intergenic regions [20]. A possible mechanism for the increase is associated with the DNA repair system [21]. Mismatches and DNA damage are proposed to be repaired exclusively by a homology-dependent recombination pathway that causes duplications, deletions or inversions with intergenic expansion, a by-product of this DNA repair system [21]. In addition, the intergenic regions expand through the acquisition of plastid DNA (ptDNA) and nuDNA sequences [19].

Nuclear DNA has also acquired mtDNA and ptDNA sequences. After finishing the Arabidopsis and rice (*Oryza sativa*) genome projects, nuDNA sequences with homology to mtDNA were found [22, 23]. Such nuclear mitochondrial DNA segments are called numts [24]. Detailed analyses of numts in Arabidopsis and rice revealed variations in their sizes and homology to the cognate mtDNA, suggesting differences in their age and mode of nuclear transfer [22]. Nine plant species containing numts that accounted for up to 10% of their genomes were reported [25], indicating that numts are ubiquitous in plant genomes. Therefore, sequence reads of numts having slight differences in their cognate mtDNA can be misinterpreted as being heteroplasmic when using NGS to detect heteroplasmy. This issue may be avoidable if a high-quality nuDNA reference is available to identify all numts that could be obstacles for heteroplasmy identification. The question remains whether numts are preserved or exhibit intraspecific polymorphism, the latter of which could be an additional obstacle for heteroplasmy identification. This issue is infrequently discussed in the study of plant heteroplasmy.

The organization of the sugar beet (*B*. *vulgaris*) mitochondrial genome was determined by chromosome walking using mtDNA samples extracted from isolated mitochondria; the integrity of each genomic clone was verified by DNA gel blot analysis prior to Sanger sequencing [26, 27]. The assembled sequence has become the reference for sugar beet mtDNA, hereafter referred to as TK-81mm-O_mt Ref. Since reference sequences determined by such old technology contain sequencing errors [28], we wanted to establish a new reference sequence for sugar beet mtDNA. TK-81mm-O_mt Ref was constructed using the TK-81mm-O sugar beet line, an old line that is no longer used in current breeding programs. Hence, we chose another sugar beet line to analyze for the new mtDNA reference. Our preliminary results of mapping NGS reads onto TK-81mm-O_mt Ref perplexed us since a number of sites were intra-individually polymorphic despite the reads emanating from the DNA of a single plant. The observed polymorphism may have represented heteroplasmy or erroneous mapping of ptDNA- or nuDNA-derived reads onto TK-81mm-O_mt Ref because of intergenomic sequence homology such as a numt. We initially wished to identify true heteroplasmy, for which a straightforward way is to exclude the intra-individually polymorphic sites on mtDNA regions with homology to ptDNA- or nuDNA from the analysis. In our study, we raised the question of whether this strategy works. Because establishing TK-81mm-O_mt Ref preceded the completion of ptDNA and nuDNA sequencing [29–31], sugar beet mtDNA regions homologous to sugar beet ptDNA or nuDNA should first be precisely determined. After that, the intra-individually polymorphic sites detected by a genome browser can be used to test whether variant callers can identify these sites. Our attempt to detect sugar beet heteroplasmy encountered serious difficulty as sugar beet lines are highly polymorphic regarding the presence or absence of numts. Intraspecific variation of numts has been less appreciated in plants but is reminiscent of findings in animals [32] and provides a lesson on the complexity of heteroplasmy study in plants.

## Materials and methods

### Plant materials

Sugar beet lines NK-195BRmm-O and NK-291BRmm-O were developed at the Hokkaido Agricultural Research Center, National Agriculture and Food Research Organization, Japan. These lines have normal fertile cytoplasm, the same cytoplasm as TK-81mm-O. NK-195BRmm-O plants were hand-emasculated before pollinating with NK-291BRmm-O. Inflorescences of F_1_ plants were enclosed in paper bags to generate the F_2_ population.

### Genome sequencing

Green leaves were collected from single plants. Total cellular DNA was isolated using a DNeasy Plant Mini Kit (Qiagen, Venlo, The Netherlands) according to the company’s instruction manual. DNA quality was checked with an Agilent 2100 Bioanalyzer (Agilent Technologies, Santa Clara, CA, U.S.A.). The sequence library was constructed using a TruSeq DNA PCR Free Library Prep Kit (Illumina, San Diego, CA, U.S.A.) (fragment size was 350 bp). HiseqX (Illumina) was used to obtain sequence reads (150 bp, paired-end). The numbers of total reads and total read bases were 941,675,126 and 142.2 Gbp for NK-195BRmm-O and 974,110,036 and 147.1 Gbp for NK-291BRmm-O, respectively. Raw data quality was checked with FASTQC v. 0.11.9 (https://www.bioinformatics.babraham.ac.uk/projects/fastqc/), a quality control tool. Nucleotide residues with a score of less than Q30 were trimmed using Sickle v. 1.33 (https://github.com/najoshi/sickle). Reads of 50 bp or shorter were excluded from further analyses.

### Read mapping onto reference genomes

Sources of reference sequences are shown in Table 1. TK-81mm-O, EL10, KWS2320 and DH1440 are sugar beet lines. M4021 is a leaf beet (chard, *Beta vulgaris*) line. Sequence reads were mapped onto reference sequences using Burrows-Wheeler Aligner (BWA) -mem v. 0.7.17 (http://bio-bwa.sourceforge.net/) [33] with default parameters to obtain SAM files that were converted into the BAM file format by the Sortsam function of Picard v. 2.27.5 (https://broadinstitute.github.io/picard/). Duplicates in the BAM file were removed using the Markduplicate function of Picard. ReadGroups were added to the BAM file by the AddOrReplaceReadGroups function of Picard.

**Table 1:** Sources of reference sequences used in this study.

Type of Assembly	Line	DDBJ/GenBank/EMBL Accession #
Mitochondrial DNA	TK-81mm-O	BA000009.3
Plastid DNA	Not provided	EF534108.1
Nuclear DNA	EL10	GCA_002917755.1
Nuclear DNA	KWS2320	GCA_000511025.2
Genomic DNA	DH1440	GCA_000510365.1
Genomic DNA	M4021	GCA_018282175.1

### Analysis of the mtDNA reference sequence

BLAST+ v. 2.13.0 (https://blast.ncbi.nlm.nih.gov/Blast.cgi?CMD=Web&PAGE_TYPE=BlastDocs&DOC_TYPE=Download) was used to analyze the TK-81mm-O_mt Ref sequence by searching for sequences homologous to the reference sequences of ptDNA, nuDNA or itself. Aligned sequences were 100 bp or longer. Boundaries of homologous sequences were determined by visual inspection of sequence alignment. Nucleotide sequences with a read depth of more than 10,000 were considered to be ptDNA homologous.

### Detection of intra-individual polymorphisms

The base composition of each site was extracted using igvtools implemented by IGV v. 2.15.2 (https://software.broadinstitute.org/software/igv/) [34] from read mapping data. Using Microsoft Excel (Microsoft Japan, Tokyo, Japan), sites containing bases distinct from TK-81mm-O_mt Ref by 1% or more of the total coverage were extracted.

### Molecular cloning and Sanger sequencing

PCR products were cloned into a plasmid vector using a TOPO TA Cloning Kit for Subcloning (Thermo Fisher Scientific, Waltham, MA, U.S.A.). Recombinant plasmids were introduced into *E*. *coli* strain DH5α, and the transformed bacteria were grown on LB plates with kanamycin [35]. Colonies with the objective DNA fragments were amplified by PCR (primers M13-Fw and M13-Rv; S1 Table). Recombinant plasmid DNA was extracted using a QIAprep Spin Miniprep Kit (Qiagen) from 2 mL cultures. Plasmid DNA was subjected to Sanger sequencing using a BigDye Terminator 3.1 Cycle Sequencing Kit (Applied Biosystems, Waltham, MA, U.S.A.) combined with an ABI3130 Genetic Analyzer (Applied Biosystems).

### Genetic segregation of DNA markers

Plant genomic DNA was isolated using a NucleoSpin Plant II Kit (Takara Bio, Kusatsu, Japan). PCR products of total cellular DNA were digested with restriction endonuclease Cla I (Takara Bio) and electrophoresed in a 2% agarose gel. R (https://www.r-project.org/) was used for statistical analyses.

### Oligonucleotide primers

Nucleotide sequences of primers are summarized in S1 Table.

### Variant callers

Freebayes v. 1.3.6 (https://github.com/freebayes/freebayes) [36], GATK v. 4.1.4.1 Haplotypecaller (https://gatk.broadinstitute.org/hc/en-us/articles/360037225632-HaplotypeCaller), samtools v. 1.16 (https://github.com/samtools/bcftools) [37], SNVer v. 0.5.3 (http://snver.sourceforge.net/manual.html), VarScan v. 2.3.8 (http://varscan.sourceforge.net/index.html) [38], and GATK v. 4.1.4.1 Mutect2 (https://gatk.broadinstitute.org/hc/en-us/articles/360037593851-Mutect2) were used. Thresholds for Freebayes and VarScan were set to 1%. Indels were filtered out from the initial VCF file using the VariantFiltration function implemented in GATK.

## Results and discussion

### Identification of unique and single-copy regions in sugar beet mtDNA

Heteroplasmy should be detected as intra-individual polymorphic sites through mapping NGS reads onto an mtDNA reference sequence, but the converse is false because shared nucleotide sequences between mtDNA and nuDNA or mtDNA and ptDNA can create similar results. Sequence differences among repeated sequences in mtDNA can also possibly cause intra-individual polymorphisms. To gain insight into the extent of these possibilities, we analyzed the composition of TK-81mm-O_mt Ref.

**Fig 1:**
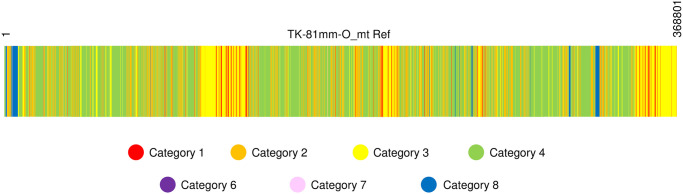
Distribution of nucleotide sequences of the eight categories in sugar beet mtDNA. TK-81mm-O_mt Ref is depicted as a rectangle spanning nucleotide positions 1 to 368801. Eight categories of sequence blocks are shown as different colors. Colors corresponding to each category are identified below.

To dissect TK-81mm-O_mt Ref, we identified three types of homologous sequences, those with homology to (a) another region of sugar beet mtDNA, (b) sugar beet nuDNA, or (c) sugar beet ptDNA (Table 2). There were 76,749 bp of repeated sequences (a homologous sequence that exists in another region(s) of TK-81mm-O_mt Ref) (the sum of categories 1, 3, 5 and 7), and the remaining regions (a total of 292,052 bp) were single-copy sequences. Nucleotide sequences homologous to nuDNA and ptDNA were 256,797 bp (the sum of categories 3, 4, 7 and 8) and 8,753 bp (the sum of categories 5, 6, 7 and 8), respectively. There were 111,759 bp of nucleotide sequences unique to TK-81mm-O_mt Ref, having no homology to either nuDNA or ptDNA (the sum of categories 1 and 2), of which 97,099 bp (category 2) were single-copy sequences. The nucleotide sequences of the eight categories are scattered throughout the TK-81mm-O_mt Ref genome to form a mosaic (Fig 1).

**Table 2:** Summary of homologous sequences in TK-81mm-O_mt Ref.

Category	Homology to			Number of nucleotides
	(a) Another region of sugar beet mtDNA	(b) sugar beet nuDNA*	(c) sugar beet ptDNA	
1	Yes	No	No	14,657
2	No	No	No	97,099
3	Yes	Yes	No	61,941
4	No	Yes	No	186,351
5	Yes	No	Yes	0
6	No	No	Yes	248
7	Yes	Yes	Yes	151
8	No	Yes	Yes	8,354
Total				368,801

* Source of the reference sequence is sugar beet line EL10.

Because completion of the TK-81mm-O_mt Ref project preceded sequence analyses of nuDNA and ptDNA, the nucleotide composition of this line had not been well known. We identified that ptDNA-homologous sequences occupy 2% of the TK-81mm-O_mt Ref genome, a value comparable to an estimate from a previous study based on the ptDNA sequence of another plant species [27]. This finding is probably due to the highly preserved gene content and nucleotide sequence of ptDNA among plant species [39]. In contrast, we found that ~70% of TK-81mm-O_mt Ref is homologous to the nuDNA of EL10. We were surprised that ptDNA-homologous sequences in TK-81mm-O_mt Ref had nuDNA counterparts in most cases (97%) (8,505 bp, the sum of categories 7 and 8); that is, mitochondrial, plastid and nuclear genomes share some nucleotide sequences. Such a complex relationship could result from successive transfer events or independent transfers of the same sequence.

### Detection of intra-individual polymorphisms in sugar beet mtDNA

To identify heteroplasmy, we focused on intra-individual polymorphic sites within the category 2 sequences (unique and single-copy sequences in sugar beet mtDNA) (Table 2). A more complex heteroplasmy is also possible when an mtDNA region homologous to ptDNA or nuDNA is heteroplasmic; however, we are currently ignoring this possibility because of the technical difficulty required to tackle this issue. We first obtained NGS reads from two Japanese sugar beet lines, NK-195BRmm-O and NK-291BRmm-O, and then mapped the reads onto the category 2 sequences. In S2 and S3 Tables, we show only sites of intra-individual polymorphism, but inter-individual polymorphism sites will be used to construct the new reference sequence in the future.

A total of 1,113 sites were found to be intra-individually polymorphic in sugar beet line NK-195BRmm-O, of which 38 sites involved indels (S2 Table). We did not consider minor alleles (represented by less than ~10 reads) because they might be artifacts. In another sugar beet line NK-291BRmm-O, 1,027 SNPs and 33 indels were present (S3 Table). Variant alleles occurred in a maximum of 9.6% of the reads. The two lines shared 720 sites.

We focused on a region encompassing 12 intra-individual polymorphic sites corresponding to positions 35,559 to 35,670 in TK-81mm-O_mt Ref. This region was located between mitochondrial genes *ccb577* and *ccb438*, both of which are involved in cytochrome *c* maturation. Differences in the nucleotide sequences among TK-81mm-O_mt Ref, NK-195BRmm-O and NK-291BRmm-O are summarized in Table 3 (the nucleotide sequences are shown in S1 Fig). For example, in TK-81mm-O_mt Ref, the nucleotide at position 3,5578 was guanine (G). Reads of NK-195BRmm-O indicated intra-individual polymorphism with either G or thymine (T) present in position 3,5578. NK-195BRmm-O and NK-291BRmm-O shared six sites. The ratios of read numbers showing variant alleles were from 1.7 to 4.4% (S2 and S3 Tables).

**Table 3:** Summary of nucleotide residues at intra-individual polymorphic sites within a 309 bp region of NK-195BRmm-O and NK-291BRmm-O.

	Nucleotide #													
	35559	35578	35581	35595	35610	35611	35613	35615	35633	35634	35653	35670	35738	35776
mt_Ref	A	G	C	G	C	C	C	G	A	C	A	G	G	G
NK-195	A/G	G/T	C	G/A	C/G	C/T	C/T	G	A/G	C	A/T	G/A	G/T	G/T
NK-291	A/G	G	C/T	G/A	C/G	C	C/T	G/A	A/G	C/T	A	G/A	G/T	G/T

Nucleotide numbers correspond to those of TK-81mm-O_mt Ref (mt_Ref). Alleles of TK-81mm-O_mt Ref (mt_Ref), NK-195BRmm-O (NK-195), NK-291BRmm-O (NK-291) are shown. Variant alleles are shown to the right.

We next examined whether these variant alleles were sequencing errors and, if not, whether they were in a coupling or repulsion phase. A 309-bp DNA fragment encompassing the 12 sites was PCR amplified from green leaves of NK-195BRmm-O and NK-291BRmm-O and cloned into plasmid vectors. We sequenced several clones and found two classes from each sugar beet line: one class has the same sequence as TK-81mm-O_mt Ref, and the other contains all variant alleles shown in Table 3. Thus, the variant alleles are in the coupling phase. These results indicate that DNA molecules having similarity with, but not identical to, TK-81mm-O_mt Ref exist in sugar beet, and this DNA sequence is polymorphic among sugar beet lines.

The IGV genome browser identified the sites showing intra-individual polymorphism (Table 3). We tested whether the same result could be reproduced by variant calling software, which would make the whole process easier if successful. Using six variant callers, we analyzed the NGS data of NK-195BRmm-O and NK-291BRmm-O to see whether the software correctly identified the sites shown in Table 3. Freebayes called 13 sites from NK-195BRmm-O; eight were matched, but five were absent (Table 4). Freebayes overlooked the remaining three sites. Haplotype callers, samtools, and SNVer called none of the sites in the 309-bp region. VarScan and Mutect2 identified nine and eight sites that were included in Table 3, respectively. However, both programs overlooked two and three sites, respectively. Similar results were obtained from the NK-291BRmm-O NGS data, except Mutect2 called eleven sites absent (Table 4). In conclusion, none of the variant callers reproduced the intra-individual polymorphic sites in the 309-bp region.

**Table 4:** Summary of variant calls in the 309-bp mitochondrial region and identity to the results of genome browsing analysis using IGV.

a) NK-195BRmm-O													
Name of software		Freebayes		Haplotype caller		Samtools		SNVer		VarScan		Mutect2	
Total number of detected sites	Number of sites present in Table 3	13	8	0	0	0	0	0	0	9	9	8	8
	Number of sites absent in Table 3		5		0		0		0		0		0
Number of overlooked sites		3		11		11		11		2		3	
b) NK-291BRmm-O													
Name of software		Freebayes		Haplotype caller		Samtools		SNVer		VarScan		Mutect2	
Total number of detected sites	Number of sites present in Table 3	14	6	0	0	0	0	0	0	8	8	20	9
	Number of sites absent in Table 3		8		0		0		0		0		11
Number of overlooked sites		5		11		11		11		3		2	

### Polymorphic presence or absence of numt among sugar beet lines

Our mtDNA sequence analysis described above referenced the chromosome-assembled sequence of U.S. sugar beet line EL10 [30]. Another chromosome-assembled reference sequence of the German sugar beet line KWS2320 [29] lacks the 309-bp sequence revealed by our BLAST search. If we had only referred to the EL10 and KWS2320 lines, our study would have concluded that the 309-bp segment was heteroplasmic, as found in previous plant heteroplasmy studies.

**Fig 2:**
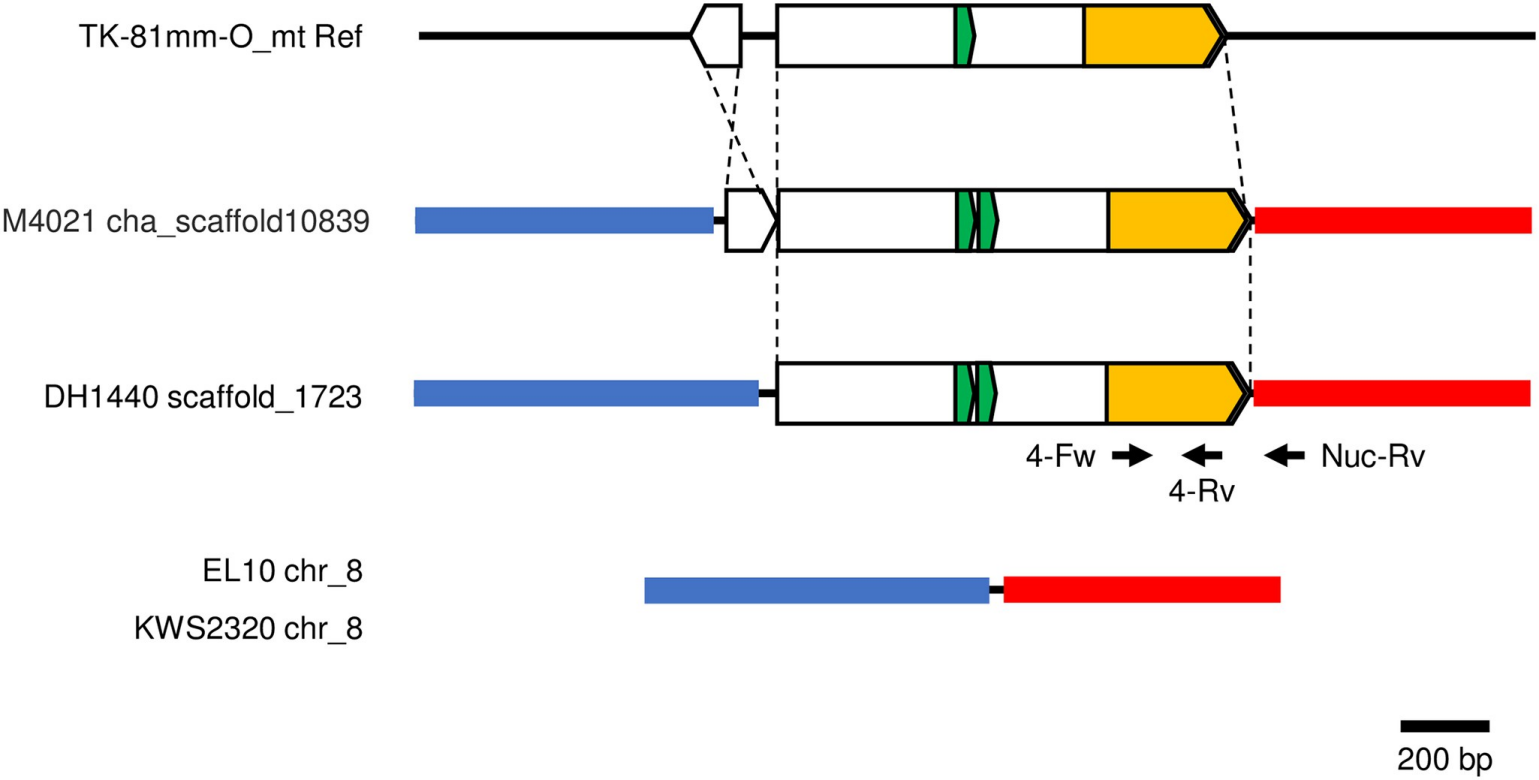
Comparison of a mtDNA segment and its related nuDNA segments in beet. Comparisons among regions of nucleotide positions 34,819 to 35,828 of TK-81mm-O_mt Ref, 36,996 to 40,435 of M4021 cha_scaffold10839 (NCBI sequence ID, JADNRI010010822.1), 22,046 to 18,959 of DH1440 scaffold1723 (KI698488.1), 28,517,680 to 28,519,310 of EL10 chromosome 8 (NC_064024.1), and 346,036 to 344,407 of KWS2320 chromosome 8 (NC_025819.2) are shown. Horizontal black lines denote unique sequences. Bold lines indicate homologous sequences whose correspondence is shown by color identity. Boxes and dashed lines indicate mtDNA-homologous sequences and their orientation, respectively. The 309-bp sequence is yellow. Horizontal black arrows indicate the positions of PCR primers.

We examined whether the 309-bp sequence existed in other sugar beet lines in addition to EL10 or KWS2320. We found that a scaffold from another German sugar beet line, DH1440 [29], contained the 309-bp sequence (Fig 2). This scaffold, DH1440_scaffold1723, has a 1,007-bp segment homologous to TK-81mm-O_mt Ref, including the 309-bp sequence (Fig 2). Compared to TK-81mm-O_mt Ref, a 42-bp duplication was found (Fig 2). The sequences surrounding the 1,007-bp segment were not homologous to TK-81mm-O_mt Ref but showed high homology to segments of chromosome 8 of EL10 and KWS2320, neither of which had mtDNA-homologous sequences (Fig 2). Organization of DH1440_scaffold1723 was found to be similar to cha_scaffold10839, which was assembled from NGS reads of M4021, a leaf beet line [40].

**Fig 3:**
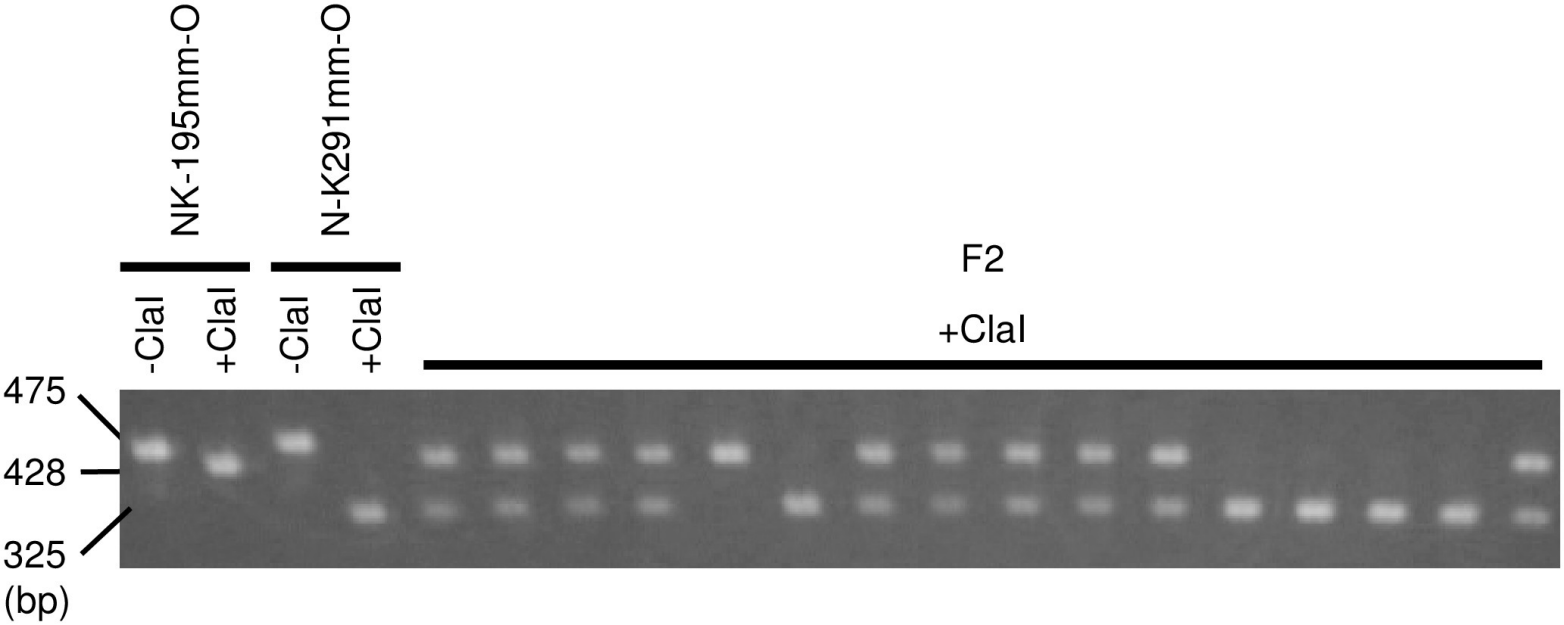
Agarose gel electrophoresis of PCR amplicons from NK-195BRm-O, NK-291BRmm-O and their F_2_. Primers 4-Fw and Nuc-Rv were used. Amplicons were electrophoresed before or after digestion with Cla I restriction endonuclease (-Cla I and +Cla I, respectively). Sizes of DNA fragments are shown on the left. The raw image of this gel is shown in S2 Fig.

We next examined whether genomes of sugar beet lines NK-195BRmm-O and NK-291BRmm-O have the same sequence arrangement as the DH1440 scaffold_1723 and M4021 cha_scaffold10839 shown in Fig 2. Two primers were prepared for this examination: 4-Fw corresponded to a mtDNA sequence and Nuc-Rv corresponded to a nuDNA (Fig 2). We obtained PCR products from both NK-195BRmm-O and NK-291BRmm-O (Fig 3).

If the amplicons were derived from nuDNA, variant alleles in the polymorphic sites should show genetic segregation. As shown in Table 3 and S1 Fig, the variant 309-bp sequences differ between NK-195BRmm-O and NK-291BRmm-O. One of the differences (nucleotide position 35,653 in Table 3) abolishes a recognition site for restriction endonuclease Cla I (**A**TCGAT to **T**TCGAT) in NK-195BRmm-O. We digested the PCR products from NK-291BRmm-O and NK-195BRmm-O with Cla I and observed a difference in their electrophoretic mobility (Fig 3), indicating that the amplicons were derived from the variant 309-bp sequence. We developed an F_2_ population from a cross of NK-195BRmm-O x NK-291BRmm-O. In the F_2_ population of 16 plants, restriction fragment length polymorphism analysis revealed one NK-195BRmm-O-type homozygous plant, ten NK-195BRmm-O-type/NK291BRmm-O-type heterozygous plants, and five NK-291BRmm-O-type homozygous plants (Fig 3). The null hypothesis for the ratio of plant numbers for each genotype is consistent with a 1:2:1 ratio, the expected ratio of nuclear gene segregation in the F_2_ generation (*p* = 0.49; Fisher’s exact test). Therefore, NK-195BRmm-O- and NK-291BRmm-O nuDNAs contain sequences homologous to the 309-bp mtDNA segment. Note that this numt could not be identified if we referenced only EL10 and might have been misinterpreted as heteroplasmy. This result also implies that numt polymorphism can explain the data claiming paternal leakage of mtDNA.

**Fig 4:**
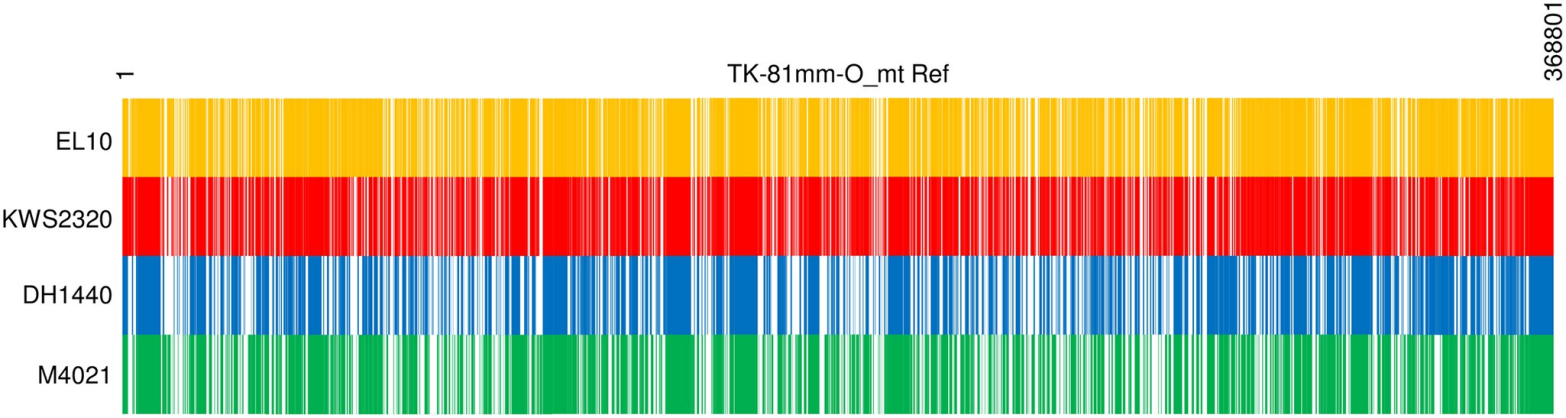
Homology to nuDNA in TK-81mm-O_mt Ref differs depending on the reference sequence. TK-81mm-O_mt Ref is shown as rectangles (numbers indicate nucleotide positions). Names of sugar beet (EL10, KWS2320, and DH1440) and leaf beet lines are shown on the left (EL10, KWS2320, DH1440 and M4021 reference sequences). Queries of 50-bp sequences were taken from TK-81mm-O_mt Ref. Those that matched the subject reference are identified as yellow, red, blue, and green vertical lines, respectively.

Intraspecific polymorphism of numts has been investigated infrequently in plants, and how it impacts plant heteroplasmy was unknown. To determine whether numt detection depends on the reference genomes used, we conducted a BLAST-based plot analysis in which every 50-bp sequence of TK-81mm-O_mt Ref was used as query and the subjects were reference sequences of sugar beet lines EL10, KWS2320, DH1440 and M4021. When a query hit the subject, we considered the query positive, and the corresponding region of TK-81mm-O_mt Ref was marked. The results are summarized in Fig 4 to show the reference-dependency of numt detection, indicating that presence or absence (P/A) polymorphism of numts exists among beet lines.

**Fig 5:**
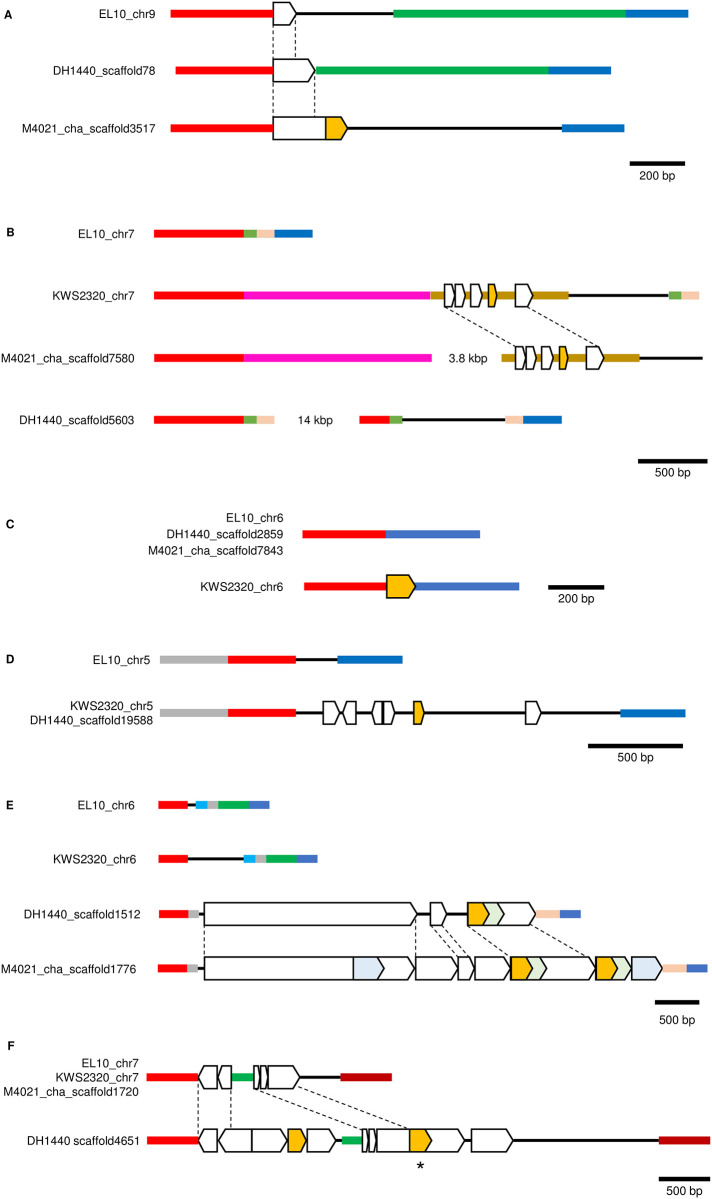
Presence or absence of numts in beet nuDNA. Numts are indicated by boxes. Yellow boxes are numts of the query sequence used for database searching. Dashed lines show numt coordination. Horizontal bars depict non-numt sequences. Homology among sequences is shown by color identity except for thin black lines, which are unique sequences. Each panel has a scale bar. **A**. Nucleotide sequence comparison of EL10 chromosome 9 (nucleotide position 17,504,107 to 17,505,958 of NC_064025.1), DH1440 scaffold78 (104,751 to 106,319 of KI697145.1), and M4021 cha_scaffold3517 (346,240 to 347,876 of JADNRI010003657.1). The corresponding locus of KWS2320 chromosome 9 is unknown because of a complex rearrangement. The query sequence was 68,238 to 68,316 of TK-81mm-O_mtDNA Ref. **B**. Nucleotide sequence comparison of EL10 chromosome 7 (51,162,715 to 51,161,579 of NC_064023.1), KWS2320 chromosome 7 (33,117,373 to 33,116,380 of CM002327.2), DH1440 scaffold5603 (48,257 to 36,467 of KI701583.1), and M4021 cha_scaffold7580 (3,999 to 4,992 of JADNRI010007566.1). Two regions are omitted but are shown as ‘14 kbp’ and ‘3.8 kbp’ in the DH1440_scaffold5603 and M4021_cha_scaffold7580, respectively. The query sequence was 241,343 to 241,452 of TK-81mm-O_mtDNA Ref. **C**. Nucleotide sequence comparison of EL10 chromosome 6 (56,357,154 to 56,356,569 of NC_064022.1), DH1440 scaffold2859 (13,701 to 13,144 of KI699428.1), M4021 cha_scaffold7843 (168,404 to 168,698 of JADNRI010008189.1), and KWS2320 chromosome 6 (51,174,328 to 51,175,018 of CM002326.2). The query sequence was 241,651 to 242,111 of TK-81mm-O_mtDNA Ref. **D**. Nucleotide sequence comparison of EL10 chromosome 5 (4,722,313 to 4,716,515 of NC_064021.1), KWS2320 chromosome 5 (46,2840,42 to 46,286,102 of CM002325.2), and DH1440_scaffold19588 (24,930 to 26,990 of KI710041.1). The M4021 scaffold covering the region shown here was not identified. The query sequence was 253,612 to 253,858 of TK-81mm-O_mtDNA Ref. **E**. Nucleotide sequence comparison of EL10 chromosome 6 (49,897,315 to 49,898,565 of NC_064022.1), KWS2320 chromosome 6 (16,480,712 to 16,478,931 of CM002326.2), DH1440_scaffold1512 (115,775 to 111,051 of KI698314.1) and M4021 cha_scaffold1776 (384,189 to 390,380 of JADNRI010001789.1). The query sequence was 285,369 to 285,865 of TK-81mm-O_mtDNA Ref. **F**. Nucleotide sequence comparison of EL10 chromosome 7 (36,164,741 to 36,160,763 of NC_064023.1), KWS2320 chromosome 7 (17,345,068 to 17,349,049 of CM002327.2), M4021 cha_scaffold1720 (190,289 to 195,270 of JADNRI010001714.1), and DH1440 scaffold4651 (221 to 7,397 of KI700858.1). The query sequences were 286,732 to 286,944 (the asterisk indicates its cognate numt) and 328,503 to 328,908 of TK-81mm-O_mtDNA Ref.

Possibly these polymorphic numts are associated with the intra-individual polymorphic sites found in NK-195BRmm-O or NK291BRmm-O. Based on Fig 4, we selected ten mtDNA regions that have homology to nuDNAs of sugar beet lines except for EL10. These ten regions were chosen to have several intra-individual polymorphic sites in NK-195BRmm-O or NK291BRmm-O and were used as query sequences for comparative analysis of EL10, KWS2320, DH1440, and M4021 genomes. For seven queries, we identified scaffolds that can be mapped onto sugar beet chromosomes (Fig 5). The remaining three queries (140,232–140,295, 146,789–146,909 and 328,233–328,284 of TK-81mm-O_mt Ref) identified three scaffolds (cha_scaffold8232, cha_scaffold4744 and cha_scaffold4475) from M4021 but mapping these scaffolds onto EL10 or KWS2320 chromosomes was very difficult because the scaffolds are mosaic with patchy homology to different chromosomal locations. More detailed analyses are necessary to identify the origin of these three scaffolds.

The identified numts are either solitary (Fig 5A and 5C) or clustered with other numts (Fig 5B and 5D–5F). In one case, two query sequences were closely mapped on the same scaffold (Fig 5F). In another case, a clustered numt spanned more than 5,000 bp in total in a locus (Fig 5E), but the sizes of single numts were generally small (average = 280 bp, mode = 59 bp and median = 94 bp). The observed numt polymorphism is explained by simple deletions in some cases, but more complex scenarios may be needed to explain the others (*e*.*g*., Fig 5B). Copy number variation of numts due to tandem duplication was observed between DH1440 and M4021 (Fig 5E). Altogether, numts absent from EL10 are frequent in sugar beet nuDNA.

We examined nucleotide residues in numts corresponding to the intra-individual polymorphic sites of NK-195mm-O and NK-291mm-O. Nucleotide sequences of the yellow-colored numts in Fig 5A–5C were identical to TK-81mm-O_mt Ref. The numts in Fig 5D and 5E and one of the numts in 5F were slightly different from TK-81mm-O_mt Ref, but the differences were so small that the relationship with the intra-individual polymorphism in the two Japanese sugar beet lines remains to be investigated. In contrast, the nucleotide sequence of the other numt in Fig 5F, identified by an asterisk, suggested the origin of some intra-individual polymorphism observed in two Japanese sugar beet lines. Table 5 shows alleles in the intra-individual polymorphic sites of NK-195BRmm-O and the corresponding residues in TK-81mm-O_mt Ref, NK-291BRmm-O and scaffold4651 of DH1440. The intra-individual polymorphism was seen in NK-195BRmm-O in these sites but not in NK-291BRmm-O (S2 and S3 Tables). The variant alleles occurred at a frequency of ~5% in NK-195BRmm-O (S2 Table). We found that the NK-195BRmm-O variant alleles perfectly matched with nucleotide residues at the corresponding sites in the DH1440 numt (S3 Fig and summarized in Table 5). In addition, the DH1440 numt had its own single nucleotide substitution and a small insertion (S3 Fig). It is possible that the DH1440-like numt exists in NK-195BRmm-O and causes the intra-individual polymorphism that could be considered as potential heteroplasmy.

**Table 5:** Summary of nucleotide residues at intra-individual polymorphic sites and those in the cognate numt.

	Nucleotide #										
	286732	286777	286781	286783	286790	286796	286816	286821	286828	286903	286944
mt_Ref	G	G	G	A	C	G	G	G	T	G	T
NK-195	G/T	G/A	G/C	A/T	C/A	G/T	G/A	G/A	T/A	G/T	T/A
NK-291	G	G	G	A	C	G	G	G	T	G	T
DH1440	T	A	C	T	A	T	A	A	A	T	A

Nucleotide numbers are aligned with those of TK-81mm-O_mt Ref (mt_Ref). Alleles of NK-195BRmm-O (NK-195), NK-291BRmm-O (NK-291) and scaffold4651 of DH1440 (DH1440) are shown. Variant alleles are shown on the right. The frequencies of variant alleles are shown in S2 and S3 Tables. The sequence comparison between TK-81mm-O_mt Ref and DH1440 scaffold4651 is shown in S3 Fig.

Numts are likely ubiquitous in plant genomes [25], but reports of their intraspecific P/A polymorphisms have not been examined in detail [41, 42]. Our results suggest that P/A polymorphism of numt among sugar beet lines is very high. The two Japanese sugar beet lines likely harbor unique numts absent from the U.S. or German lines. Our study shows that such lineage-specific numts can be erroneously seen as examples of heteroplasmy. Therefore, a single representative reference is insufficient for a plant heteroplasmy study if the researcher is dealing with several lines. Note that previous plant heteroplasmy studies did not consider this point. In the green leaves of sugar beet, the copy number ratio of mtDNA to nuDNA was estimated to be 40:1 to 60:1 [43]: hence, approximately 2% of reads mapped onto TK-81mm-O_mtDNA Ref will be nuDNA in origin if the cognate numt exists. The frequencies of variant alleles in S2 and S3 Tables are similar to the read ratios of numt and mtDNA.

The ratio of true heteroplasmy in the detected intra-individual polymorphic sites is unknown but could be determined after simultaneous *de novo* sequence assembly of mtDNA, ptDNA and nuDNA at the genome level in single plants of NK-195mm-O and NK-291mm-O. Software to identify intraindividual polymorphism efficiently will be necessary as the current variant callers are inadequate. Therefore, we propose that identifying heteroplasmic sites from intra-individual polymorphic sites is technically infeasible at present since excluding numts is extremely difficult. Considering that sequence transfer of ptDNA segments to nuDNA occurs at the laboratory scale [44], and if this is also the case for mtDNA transfer to nuDNA to be a source of new numts, detection of variant mtDNA-like sequences is too early to label as heteroplasmic without further supporting evidence.

## Conclusion

We stress that we do not intend to advocate the absence of heteroplasmy in sugar beet but, rather, the difficulty in heteroplasmy detection through NGS data. Caveats in our analysis include: we did not consider reads with a depth of less than 1%, did not consider the possibility of heteroplasmic sites within homologous sequences to ptDNA or nuDNA, and did not consider ectopically recombined mtDNA molecules. Our data indicate that intra-individual polymorphism of mtDNA that is detected as SNPs or indels should first be treated as from numt origin. Importantly, heteroplasmy detection from diverse plant materials should not rely on a single representative reference nuclear genome.

P/A polymorphism of numts within a species is well documented in vertebrates (*e*.*g*., [45]). Together with two previous reports [41, 42], our data suggest P/A polymorphism of numts is also the case in plants. Unfortunately, the pretreatments of DNA samples for vertebrate heteroplasmy detection, such as PCR amplification of the entire mtDNA region or enrichment of the mitochondrial fraction, [2] are not always feasible analyses for plants. The whole picture of numt polymorphism in plants is difficult to obtain because the plant mitochondrial genome is much larger compared to vertebrates and shows extensive organizational differences within a species [19]. Scarcelli et al. [46] proposed a way to examine plastid heteroplasmy from NGS data but mentioned the difficulty in similarly examining plant mitochondria. A procedure to detect mitochondrial heteroplasmy from NGS data must be established, including software development, to resolve these issues fully.

## Supporting information

S1 Table Nucleotide sequences of primers used in this study. (XLSX)

S2 Table Count details of intra-individually polymorphic sites detected from NK-195mm-O. (XLSX)

S3 Table Count details of intra-individually polymorphic sites detected from NK-291mm-O. (XLSX)

S1 Fig Nucleotide sequence of a 309-bp region belonging to category 2. This sequence corresponds to nucleotide positions 35,507 to 35,815 of TK-81mm-O_mt Ref (Ref). Two classes of DNA molecules were found from NK-195BRmm-O (195) and NK-291BRmm-O (291): those identical to TK-81mm-O_mt Ref (suffixed by _R) and those having variant alleles (_V). Positions of PCR primers (4-Fw and 4-Rv) are shown by lowercase letters. Asterisks indicate sites with variant alleles. Variant alleles are shown in red. The recognition site of the Cla I restriction endonuclease is underlined.
(DOCX)

S2 Fig Raw image of the agarose electrophoresis gel shown in Fig 4. (PPTX)

S3 Fig Comparison of nucleotide sequences between TK-81mm-O_mt Ref and scaffold4651 of DH1440 (DDBJ/GenBank/EMBL accession number KI700858). Nucleotide numbers are in accordance with those in the database. Different residues in DH1440 are shown with red letters. Dashes are incorporated for maximum matching.
(DOCX)
