# Supplementary material for: Nuclear DNA segments homologous to mitochondrial DNA are obstacles for detecting heteroplasmy in sugar beet (Beta vulgaris L.)
Source: PLoS One. 2023 Aug 8;18(8):e0285430. doi: 10.1371/journal.pone.0285430 (PMC10409277; doi:10.1371/journal.pone.0285430)
Supplement: S1 Fig — This sequence corresponds to nucleotide positions 35,507 to 35,815 of TK-81mm-O_mt Ref (Ref). Two classes of DNA molecules were found from NK-195BRmm-O (195) and NK-291BRmm-O (291): those identical to TK-81mm-O_mt Ref (suffixed by _R) and those having variant alleles (_V). Positions of PCR primers (4-Fw and 4-Rv) are shown by lowercase letters. Asterisks indicate sites with variant alleles. Variant alleles are shown in red. The recognition site of the Cla I restriction endonuclease is underlined. (DOCX) [file pone.0285430.s004.docx]

4-Fw

Ref gtgaatcagtccgaaatgctatggATAGATAGTTTTAGCCAATATCGATCCGATCGGGAA 35566

195_R gtgaatcagtccgaaatgctatggATAGATAGTTTTAGCCAATATCGATCCGATCGGGAA 60

291_R gtgaatcagtccgaaatgctatggATAGATAGTTTTAGCCAATATCGATCCGATCGGGAA 60

195_V gtgaatcagtccgaaatgctatggATAGATAGTTTTAGCCAATATCGATCCGGTCGGGAA 60

291_V gtgaatcagtccgaaatgctatggATAGATAGTTTTAGCCAATATCGATCCGGTCGGGAA 60

*

Ref TGAGAGTACCTGACCTCTTGCCTATTCAGCAGTATTTATTATACCGCCGCACTCTTTATA 35626

195_R TGAGAGTACCTGACCTCTTGCCTATTCAGCAGTATTTATTATACCGCCGCACTCTTTATA 120

291_R TGAGAGTACCTGACCTCTTGCCTATTCAGCAGTATTTATTATACCGCCGCACTCTTTATA 120

195_V TGAGAGTACCTTACCTCTTGCCTATTCAACAGTATTTATTATAGTGTCGCACTCTTTATA 120

291_V TGAGAGTACCTGACTTCTTGCCTATTCAACAGTATTTATTATAGCGTCACACTCTTTATA 120

* * * ** * *

Ref GTCGCGACTGTTGTCATGGAAAAAAAATCGATTTTCTGTCAAAGAATCATGCTTAACACA 35686

195_R GTCGCGACTGTTGTCATGGAAAAAAAATCGATTTTCTGTCAAAGAATCATGCTTAACACA 180

291_R GTCGCGACTGTTGTCATGGAAAAAAAATCGATTTTCTGTCAAAGAATCATGCTTAACACA 180

195_V GTCGCGGCTGTTGTCATGGAAAAAAATTCGATTTTCTGTCAAAAAATCATGCTTAACACA 180

291_V GTCGCGGTTGTTGTCATGGAAAAAAAATCGATTTTCTGTCAAAAAATCATGCTTAACACA 180

** * *

Ref GCCTATAGCGTAAAGGCATTCGAACCGCGTCTAAACTAAATTAAGGGGAAGGCCCGTATT 35746

195_R GCCTATAGCGTAAAGGCATTCGAACCGCGTCTAAACTAAATTAAGGGGAAGGCCCGTATT 240

291_R GCCTATAGCGTAAAGGCATTCGAACCGCGTCTAAACTAAATTAAGGGGAAGGCCCGTATT 240

195_V GCCTATAGCGTAAAGGCATTCGAACCGCGTCTAAACTAAATTAAGGGGAAGTCCCGTATT 240

291_V GCCTATAGCGTAAAGGCATTCGAACCGCGTCTAAACTAAATTAAGGGGAAGTCCCGTATT 240

*

Ref CTCTCTTCTGTAGATACGCTATCCTTTCTGGCTATGCTATGGGggaaaggaagtgagatc 35806

195_R CTCTCTTCTGTAGATACGCTATCCTTTCTGGCTATGCTATGGGggaaaggaagtgagatc 300

291_R CTCTCTTCTGTAGATACGCTATCCTTTCTGGCTATGCTATGGGggaaaggaagtgagatc 300

195_V CTCTCTTCTGTAGATACGCTATCCTTTCTTGCTATGCTATGGGggaaaggaagtgagatc 300

291_V CTCTCTTCTGTAGATACGCTATCCTTTCTTGCTATGCTATGGGggaaaggaagtgagatc 300

*

4-Rv

Ref tacagattg 35815

195_R tacagattg 309

291_R tacagattg 309

195_V tacagattg 309

291_V tacagattg 309

Fig. S1. Nucleotide sequence of a 309-bp region belonging to category 2. This sequence corresponds to nucleotide position of 35507 to 35815 of TK-81mm-O_mt Ref (Ref). Two classes of DNA molecules were found from NK-195BRmm-O (195) and NK-291BRmm-O (291): those identical to TK-81mm-O_mt Ref (suffixed by _R) and those having variant alleles (_V). Positions of PCR primers (4-Fw and 4-Rv) are shown by lower case letters. Sites with variant alleles are indicated by asterisks. Variant alleles are red-colored. Recognition site of Cla I restriction endonuclease is underlined.
