# Supplementary material for: Nuclear DNA segments homologous to mitochondrial DNA are obstacles for detecting heteroplasmy in sugar beet (Beta vulgaris L.)
Source: PLoS One. 2023 Aug 8;18(8):e0285430. doi: 10.1371/journal.pone.0285430 (PMC10409277; doi:10.1371/journal.pone.0285430)
Supplement: S2 Fig — (PPTX) [file pone.0285430.s005.pptx]

## Slide 1
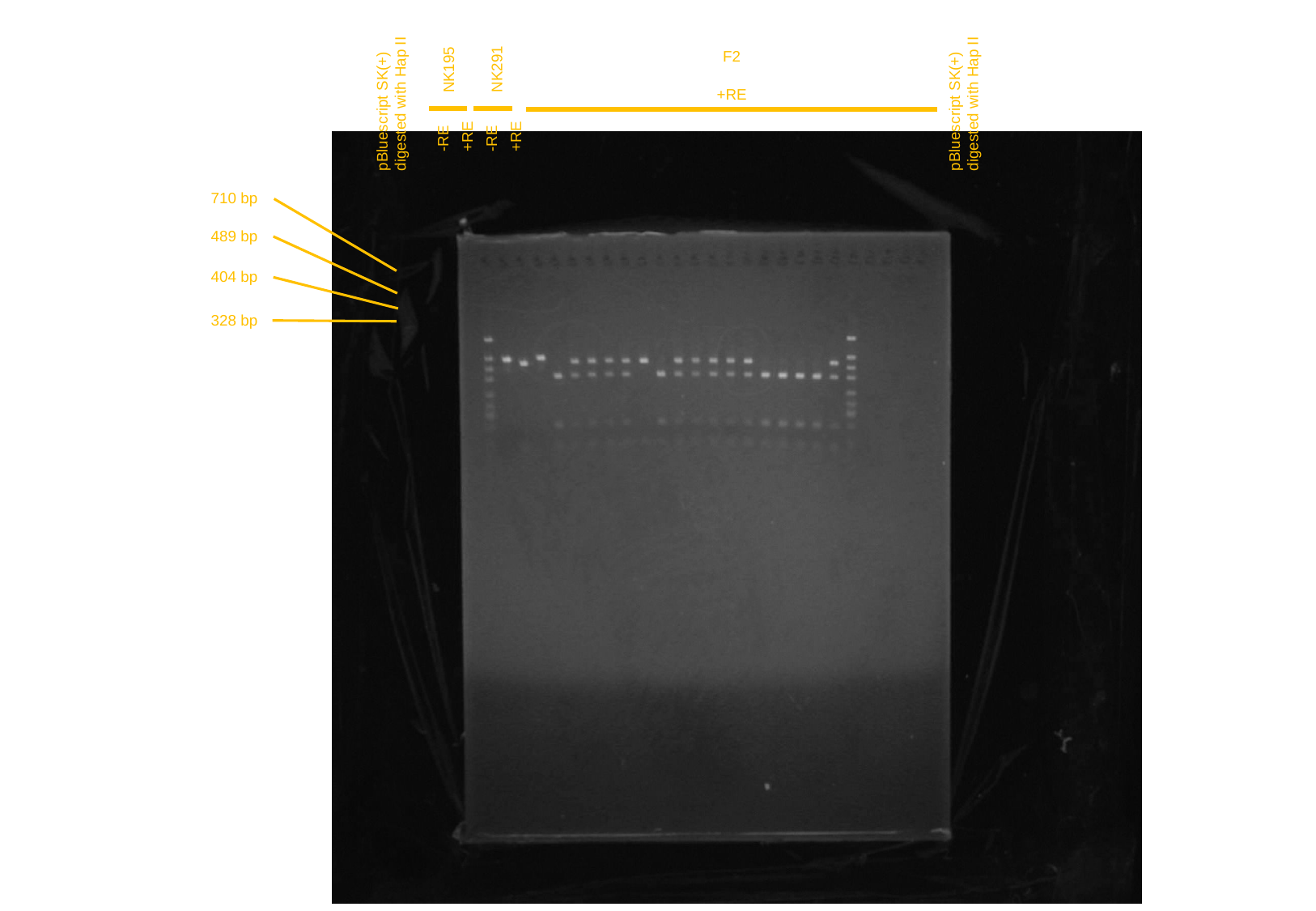

pBluescript SK(+)
digested with Hap II
pBluescript SK(+)
digested with Hap II
710 bp
489 bp
404 bp
328 bp
F2
NK291
NK195
+RE
-RE
+RE
-RE
+RE
