# Supplementary material for: Nuclear DNA segments homologous to mitochondrial DNA are obstacles for detecting heteroplasmy in sugar beet (Beta vulgaris L.)
Source: PLoS One. 2023 Aug 8;18(8):e0285430. doi: 10.1371/journal.pone.0285430 (PMC10409277; doi:10.1371/journal.pone.0285430)
Supplement: S3 Fig — Nucleotide numbers are in accordance with those in the database. Different residues in DH1440 are shown with red letters. Dashes are incorporated for maximum matching. (DOCX) [file pone.0285430.s006.docx]

mt_Ref TGACACGCGGA---T-GCATTTCCTTTTATCCGTAGAACTTCTAACCAAAAGATTCATTC 286756

DH1440 TGACACGCGGA**TCG**T**G**GCATTTCCTTTTATCCGTA**T**AACTTCTAACCAAAAGATTCATTC 3276

mt_Ref TTTATCAAGTACCATTCAAAGGTTGCATTATCTCTTTGAGTGAAGAAGAGAAGGACTTTG 286816

DH1440 TTTATCAAGTACCATTCAAA**A**GTT**C**C**T**TTATCTATTTGA**T**TGAAGAAGAGAAGGACTTT**A** 3336

mt_Ref TATAGACACTCTTGAGCCATGTTCGTTGCCCTAGGCTCACTACActattatttaattgaa 286876

DH1440 TATA**A**ACACTC**A**TGAGCCATGTTCGTTGCCCTAGGCTCACTACACTATTATTTAATTGAA 3396

mt_Ref ttcTATTGATAGATTCTAGCTCCAAAGAACATATACATGGAGCTATGTCGACTTACGTAC 286936

DH1440 TTCTATTGATAGATTCTAGCTCCAAA**T**AACATAT**T**CATGGAGCTATGTCGACTTACGTAC 3456

mt_Ref GTACGTCTCGTTGA 286950

DH1440 GTACGTC**A**CGTTGA 3470

Fig. S3 Comparison of nucleotide sequences between TK-81mm-O_mt Ref and scaffold4651 of DH1440 (DDBJ/GenBank/EMBL accession number KI700858). Nucleotide numbers are in accordance with those in the database. Different residues in DH1440 are shown by red letters. Dashes are incorporated for maximum matching.
